# Axillary Management: How Much Is Too Much?

**DOI:** 10.1007/s11912-024-01539-0

**Published:** 2024-05-15

**Authors:** Nicci Owusu-Brackett, Benjin Facer, Dionisia Quiroga, Ashley Pariser, Michael Grimm, Sasha Beyer, Sachin Jhawar, Bridget A. Oppong

**Affiliations:** 1 Division of Surgical Oncology, Department of Surgery, The Ohio State University Wexner Medical Center and James Cancer Hospital, Columbus, OH USA; 2 Department of Radiation Oncology, The Ohio State University Wexner Medical Center and James Cancer Hospital, Columbus, OH USA; 3 Division of Medical Oncology, Department of Internal Medicine, The Ohio State University Wexner Medical Center and James Cancer Hospital, Columbus, OH USA; 4 West Virginia University School of Medicine, Morgantown, WV USA

**Keywords:** Breast Cancer, Surgery, Axilla, Neoadjuvant Systemic Therapy, Nodal Irradiation

## Abstract

**Purpose of Review:**

To review the current management of the axilla in breast cancer.

**Recent Findings:**

Axillary dissection is no longer indicated in patients with clinically node-negative axilla with 1–2 positive sentinel lymph nodes following upfront surgery or in patients with clinically node-negative axilla following neoadjuvant chemotherapy.

**Summary:**

Breast cancer has evolved away from routine axillary clearance to the less invasive sentinel lymph node biopsy to now complete omission of axillary sampling in select patients. We will review the most salient evidence that has shaped these practice changes over the last three decades. Current practice controversies are especially relevant for elderly populations and those receiving neoadjuvant therapy. Ongoing clinical trials will provide data to further guide breast cancer surgical management.

## Introduction

Breast cancer is the most diagnosed cancer in U.S. women affecting 1 in 8 women and the 2nd leading cause of cancer death [1]. In 2022, 287,850 invasive cancers and 51,400 non-invasive (in situ) cancers were diagnosed, including 2710 cases in men [1]. Breast cancer survival is high with five-year survival around 90% [1]. However, five-year survival remains lower for African American women (78%) compared to White counterparts (92%) [1]. Despite the disparities in outcomes, all groups are experiencing improvement in breast cancer survival due to advancements in management across the modalities of surgery, radiation, and medical therapies. As a result, the impact of axillary management on breast cancer survival has been evaluated.

### Breast Cancer Surgery

Historically, management of breast cancer in the axilla was the same as the in-breast disease in the 1890s. Halsted developed the radical mastectomy which involved resection of the breast, both pectoralis muscles and axillary lymph nodes within levels I, II and III [2]. In 1948, Patey modified the radical mastectomy with sparing of the pectoralis major, which was further modified by Auchincloss in 1950 with additional preservation of the pectoralis minor [3, 4]. Moreover, this modification by Auchincloss brought about the first de-escalation of axillary management with preservation of level III axillary lymph nodes. Studies support this de-escalation given that only 1–3% of stage I—II patients without involvement of level I or II nodes have positive level III nodes [5].

Even with the modified radical mastectomy, significant morbidity issues remain including lymphedema, paresthesia, and post-operative pain [6]. Due to the morbidity associated with axillary lymph node dissections (ALND), questions regarding the benefit of this maximally invasive surgery arose. National Surgical Adjuvant Breast and Bowel Project (NSABP)B-04 evaluated the benefit of ALND on survival for patients with palpable, non-fixed, operable tumors in the breast and axilla only [6]. No significant differences in disease-free or overall survival were noted between patients who underwent radical mastectomy, total mastectomy, or total mastectomy with regional irradiation.

The concept of sentinel lymph node biopsy (SLNB) was introduced by Giuliano et al., in 1994 at the John Wayne Cancer Institute [7]. It correctly identified nodal status in 95% of patients in comparison to axillary dissection when using blue dye alone. Radiotracer was later introduced and today most surgeons use both blue dye and radiotracer (dual tracer). NSABP B-32 evaluated the feasibility and safety of SLNB compared to ALND and revealed high sentinel node detection and accuracy with false-negative rates (FNR) of 0% – 15% [8]. In addition, SLNB was associated with less morbidity including decreased pain, fewer seromas, and less lymphedema, as well as better range of motion and quality of life compared to patients who underwent ALND [9, 10]. The reduction in lymphedema rates from 15–40% to 1–3% after SLNB made adoption of this technique attractive to surgeons.

### Upfront Surgery

**Fig. 1:**
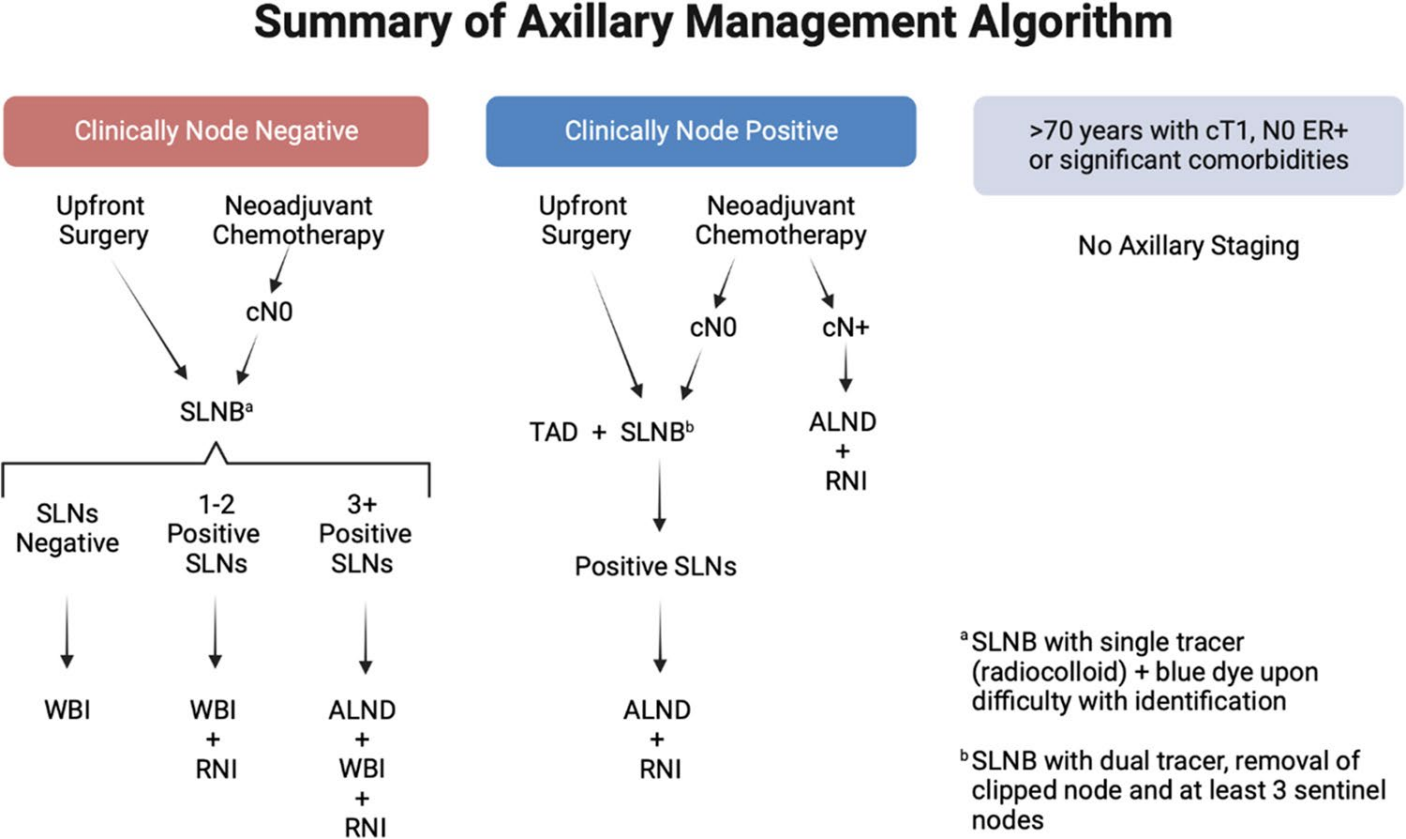
Summary of Axillary Management Algorithm

For most patients, the first definitive breast cancer therapy is surgery in the form of partial or complete mastectomy. Surgical decision making is based on the extent of disease in the breast, multicentricity, extent of calcifications, and the ability to safely deliver radiotherapy [11–13]. Historically, biologic factors such as tumor histology, grade, and estrogen receptor, progesterone receptor, and HER2 status did not factor into deciding between breast-conserving therapy and mastectomy [12, 13]. NSABP B-04 showed no significant difference in survival between women treated with the Halsted radical mastectomy and those treated with less extensive surgery, while B-06, showed that with lumpectomy and postoperative breast irradiation, outcomes were comparable to mastectomy [6, 14]. Current guidelines recommend axillary staging with SLNB for patients with early-stage breast cancer under the age of 70 and without significant comorbidities (Fig. 1).

#### Clinically Node-Negative Disease

For early-stage breast cancer patients with clinically negative axilla undergoing upfront surgery, SLNB with single tracer (radiocolloid) is standard of care with the addition of a second tracer (blue dye) upon difficulty identifying the SLNs. The SENTINA trial noted no difference in identification rates with dual tracer compared to single radiocolloid tracer (99.5% *v* 98.8%, *P* = not reported) [6].

Per the American College of Surgeons Oncology Group (ACOSOG) Z0011 trial, patients with 1–2 positive sentinel lymph nodes undergoing BCT or total mastectomy do not require an axillary dissection. This study found that patients with cT1-2 breast cancer and 1–2 positive sentinel nodes following breast-conserving surgery with planned whole breast radiation therapy (WBRT) had no overall or disease-free survival benefit when undergoing ALND compared to the observation cohort [15]. In addition, it was noted that 27% of patients who underwent ALND had cancer in non-sentinel lymph nodes. This suggests that some of the patients who underwent SLNB alone may have had residual non-sentinel node disease that was not resected, which did not result in a significantly worse survival [16]. To prevent overtreatment, patients with negative sentinel lymph nodes should not undergo ALND [17]. The ACOSOG Z0011 findings also call into question the role of ultrasound in the pre-operative evaluation of the axilla [18]. Patients with clinically negative nodes do not have to undergo an axillary ultrasound and yet many routinely perform an ultrasound at diagnosis. This is suboptimal as a positive axillary ultrasound would unnecessarily commit many patients to ALND who would otherwise meet criteria for omission. [19, 20]. However, there is evidence that disease identified by axillary ultrasound suggests a higher axillary disease burden compared to the disease identified by SLNB. In cases where neoadjuvant chemotherapy is being considered, evaluation of nodal involvement and biopsy confirmation would impact management [21].

The Z0011 trial also produced some controversies with radiotherapy after lumpectomy. A subsequent analysis of the Z0011 trial demonstrated that 43 (18.9%) of patients received directed regional nodal RT using greater than or equal to three fields: 22 in the ALND arm and 21 in the SLND arm [21]. Therefore, more of the patients than originally intended received radiation to the lymph nodes. These findings suggested that residual minimal axillary disease defined as 2 or fewer macrometastases were sufficiently treated with adjuvant therapies such as whole breast radiation, chemotherapy and/or endocrine therapy. Similarly, the International Breast Cancer Study Group randomized clinical trial (IBCSG 23–01) showed that patients with cT1-2 breast cancer and 1–2 positive sentinel nodes undergoing breast conservation surgery or total mastectomy also did not derive a disease-free survival benefit at 10 years from ALND compared to observation [22]. While patients who underwent total mastectomy only comprised 9% of the study population, these findings suggested ALND may not be required for total mastectomy patients with minimal axillary disease of 1–2 positive nodes. As a result, the current available evidence supports omission of ALND for patients undergoing breast conserving therapy or total mastectomy with 2 or fewer positive sentinel lymph nodes without gross extranodal disease due to the efficacy of complementary therapies such as chemotherapy and radiation; however, questions arose regarding whether axillary RT could replace ALND.

#### Adjuvant RT

Standard of care adjuvant radiotherapy following breast-conserving surgery is whole breast irradiation (WBI) or in select cases accelerated partial breast irradiation (APBI). Post-mastectomy radiotherapy (PMRT) focuses radiation at the chest wall and regional lymph node basins. While the decision regarding adjuvant radiotherapy is multifactorial, patients with positive lymph nodes receive WBI with regional nodal irradiation (RNI) or PMRT to decrease the risk of regional recurrence or distant metastases [23, 24]. Typically, RNI is not offered in patients without proven or a high suspicion of nodal involvement. Select patients with medial/central tumors or triple negative breast cancer (TNBC), locoregional irradiation may be considered [25, 26••]. The National Cancer Institute of Canada (NCIC) MA.20 trial evaluated the role of RNI in addition to whole breast radiation in patients undergoing conservation surgery with SLNB or ALND [16]. 85% of patients within this trial had 1–3 positive nodes, and 5% had greater than 4 positive nodes. 10% of the patients were node-negative with high-risk features such as T3, T2 with fewer than 10 lymph nodes removed and grade 3, estrogen receptor-negative disease or lymphovascular invasion. The MA.20 trial demonstrated that RNI resulted in improved DFS (disease-free survival) (estrogen receptor–negative [ER–] 61.6% *v* 76.2, HR 0.56, 95% CI 0.39–0.81, *P* = 0.04; progesterone receptor–negative [PR–] 70.5% *v* 81.9%, HR 0.57, 95% CI 0.41–0.80; *P* = 0.03) and distant DFS (86.3% RNI group *v* 82.4% WBI group, HR 0.76, 95% CI 0.60–0.97, *P* = 0.03) at 10 years with the addition of RNI in all patients with high-risk features [23].

Regarding the need for regional nodal irradiation (RNI), the After Mapping of the Axilla: Radiotherapy or Surgery (AMAROS) trial demonstrated no difference in the 10-year axillary recurrence rate (1.82% v 0.93%, P = 0.37) as well as no survival benefit at 10 years (84.6% v 81.4%, P = NS) between RNI compared to ALND in a population of cT1/2N + patients with predominantly low volume axillary disease following breast-conserving surgery [27, 28]. A lower lymphedema risk at 5 years (11% v 23%, P < 0.0001) was observed with radiotherapy. Similarly, the MA.20 study showed no survival benefit at 10 years with the addition of RNI to whole breast radiation (HR 0.91, 95% CI 0.72–1.13; *P* = 0.38) [23]. An improved DFS (HR 0.76, 95% CI 0.61–0.94; *P* = 0.01) was noted with RNI added to WBI but an increase in pneumonitis and radiation dermatitis (1.2% *v* 0.2%, *P* = 0.01; 49.5% *v* 40.1%, *P* < 0.001, respectively) was observed as well. The European Organisation for Research and Treatment of Cancer (EORTC) 22,922/10925 trial also showed a 10-year DFS benefit to the addition of nodal XRT (72.1% v 69.1%, P = 0.04) but no overall survival benefit (82.3% v 80.7%, P = 0.06) [26••]. However, given the improved local and distant recurrence, post-mastectomy radiation is recommended for patients with minimal axillary disease [29, 30].

For patients with a large burden of axillary disease defined as 3 or greater positive sentinel nodes, ALND and local regional nodal irradiation remain the standard of care as these populations have not been well studied.

#### Clinically Node-Positive

Patients with clinically palpable axillary lymph nodes should undergo axillary ultrasound with biopsy and clip placement to determine nodal involvement. For patients without pathologic evidence of disease undergoing upfront surgery, SLNB as described above is preferred while those with pathologic positivity undergoing upfront surgery should have an ALND.

### Surgery Post-Neoadjuvant Chemotherapy

Advances in other treatment modalities also help to change practice. Starting in the 1970s, neoadjuvant chemotherapy (NAC) is now utilized in over 20% of breast cancer cases, particularly in the setting of a large primary tumor or clinically positive lymph nodes (cN +) [30, 31]. The potential benefits of NAC include: improved operability (downstaging of breast and axillary disease leading to de-escalation of surgery), improved cosmesis (decreased size of necessary surgical resection), acquisition of valuable prognostic information based on response status, avoidance of axillary lymph node dissection (ALND) and the ability to personalize adjuvant systemic therapy [31, 32]. In the modern era, due to advances in systemic therapy, decreasing the use of ALND is becoming more feasible. NSABP B-18 showed that neoadjuvant chemotherapy (NAC) provided the same survival benefit to patients as adjuvant chemotherapy [32]. Furthermore, NSABP B-27 showed that a significant proportion of patients achieve a clinical and pathologic complete response following NAC. These clinical and pathologic responses allow for use of less-invasive surgical techniques such as breast conserving surgery or SLNB; however, identification of the appropriate patients is integral [33–35].

Neoadjuvant systemic therapy can also provide prognostic information based on response to therapy. Patients with triple-negative and HER2-positive breast cancer have a high likelihood of response to NAC with pathologic complete response (pCR) rates up to 46% and 65%, respectively [36, 37••, 38]. There is a strong correlation between these pathologic responses to neoadjuvant therapy and long-term survival outcomes for these patients with early-stage breast cancer [36, 39]. Moreover, in TNBC and HER2-positive breast cancer cases where a pCR is not achieved with neoadjuvant therapy, escalation of adjuvant therapy can be offered to improve survival rates (capecitabine, TDM-1, olaparib, respectively) [40–42]. As a result, preoperative systemic therapy is recommended in patients with operable tumors with cT1c and/or cN + or TNBC or HER2-positive disease. In addition, NAC is preferred in patients with large operable tumors relative to breast size who desire BCS and patients with inoperable tumors, with inflammatory breast cancer or with cT4 tumors. Breast MRI is recommended prior to initiation of NAC to assist with assessment of response.

For patients with good response who are clinically node-negative after NAC, SLNB should be performed [6].

#### SLN Technique

Following NAC, false-negative rates of SLNB are commonly higher than the predetermined acceptable threshold of 10%. Surgical studies have focused on methodology to obtain an adequate false-negative rate (FNR) of 10%. Current recommendations in the post-NAC setting include use of dual tracer, radiocolloid and blue dye, in addition to a targeted axillary dissection which consists of removal of the clipped metastatic node and removal of at least 3 sentinel nodes to obtain an acceptable FNR. The SN Biopsy Following Neoadjuvant Chemotherapy (SN FNAC) trial had an overall FNR of 8.4% in patients with T0-3N1-2 disease [43]. The FNR was noted to be 16% when single tracer (radiocolloid) was used while use of dual tracer resulted in a decrease of the FNR to 5.2%. In addition, a FNR of 18.2% was noted when only one sentinel node was removed but decreased to 4.9% when 2 or more sentinel nodes were removed. The SENTINA trial reported an overall FNR of 14.2% in patients with clinically node positive disease that converted to clinically node negative disease following NAC [6]. Use of dual tracer resulted in a FNR of 8.6% from 16% while removal of 3 SLNs led to a FNR of 7.3% from 18.5% with 2 nodes. Finally, ACOSOG Z1071 similarly demonstrated improvement of the FNR rates to 10.8% with dual tracer and 9.1% with removal of at least 3 nodes in patients with clinically node positive disease [44, 45].

In 2016, Caudle et al. demonstrated a FNR of 10% with removal of SLNs only, which decreased to 4% with removal of pre-NAC clipped metastatic nodes [46]. It was noted that 23% of clipped nodes were not SLNs. Finally, a FNR of 2% was obtained with removal of the I-125 seed localized clipped metastatic node and SLNs in a technique known as targeted axillary dissection (TAD). Targeted axillary dissection is currently the preferred method of axillary staging for patients who presented with cN1 disease that converted to cN0 following NAC. Of note,

NSABP B-32 demonstrated that FNR doesn’t translate to local recurrence [47]. However, studies have shown that LR rates are generally low, justifying the de-escalation of axillary management [10].

#### cN1 Conversion to cN0 Following NAC

If the clipped node and SLNs are negative, RNI is controversial, but typically recommended. NSABP B-51 is an ongoing study evaluating the efficacy of nodal irradiation in cT1-3 cN1 patients with no residual nodal disease following NAC [48•]. The primary endpoint is invasive breast cancer recurrence-free interval while secondary endpoints include overall survival, locoregional recurrence-free interval and distant recurrence-free interval. As we aim to improve patient quality of life, studies have also been designed to evaluate the accuracy of post-NAC tumor bed biopsies in patients with complete clinical response to assess for residual disease. These biopsies may help assess if patients can have definitive RT and avoid surgery completely. In a single-institution MD Anderson study, researchers used fine needle aspiration and vacuum-assisted core biopsy to determine residual disease and found a negative predictive value of 95% [49]. Their subsequent phase II trial omitted surgical axillary staging in lieu of RT in patients with complete clinical and pathologic response following NAC [50]. Patients received WBI and boost and were noted to have a locoregional recurrence of 0% in 31 patients after 26 months of follow up. While more long-term follow up is needed, this study suggests that surgical axillary staging may be omitted in certain low-risk populations due to the efficacy of NAC and radiation as well as the increasing use of radiosensitizing agents such as capecitabine, Trastuzumab emtansine, Olaparib and Abemaciclib. Future studies are needed to further evaluate these interesting findings. Furthermore, tools such as circulating tumor DNA levels after NAC may provide prognostic information of recurrence risk and survival and may even help identify patients who may or may not benefit from adjuvant RT [47, 48•, 49–54, 55••, 56].

If the clipped node or SLNs are positive, an axillary dissection is necessary, along with RNI. There is a trial, Alliance A011202, which investigated the role of RNI in patients with cN1 disease who have a positive SLNB following NAC [54]. The trial is closed to accrual and results pending. The study will randomize patients to completion ALND with WBI and RNI vs WBI and RNI alone to answer whether these patients can undergo RT alone and omit completion ALND without affecting their invasive breast cancer RFI and overall survival.

#### cN1 following NAC

Patients with suboptimal response who remained clinically node-positive following NAC require an ALND with adjuvant locoregional radiation therapy due to chemo-resistant disease and high risk of recurrence and metastases.

### No Axillary Staging Necessary

The goal of axillary staging with SLNB is to help guide decisions regarding adjuvant therapy [55••]; however, this isn’t always achieved. A meta-analysis evaluated whether axillary staging in elderly breast cancer patients impacted outcomes [56]. The two studies included showed that for elderly women over the age of 70 years with early-stage cT1-2 cN0 breast cancer there was no survival benefit derived from ALND (RR 0.99, 95% CI 0.79–1.24, *P* = 0.92 [57, 58]. The omission of ALND resulted in no difference in their breast cancer-specific mortality either (relative risk [RR] 1.07, 95% CI 0.72–1.57, *P* = 0.75) but did result in an increase in regional recurrence risk (RR 0.24, 95% CI 0.06–0.95, *P* = 0.04). Due to the increased risk of regional recurrence, the Society of Surgical Oncology Choosing Wisely guidelines apply the recommendation of omission of axillary staging with SLNB to only low-risk hormone receptor-positive elderly patients especially given the efficacy of adjuvant hormonal therapy [59]. The results of the Cancer and Leukemia Group B (CALGB) 9343 trial supported this recommendation as low rates of axillary recurrences (3%) were observed for > 70 years old women with early-stage breast cancer who received tamoxifen but did not undergo axillary staging [60]. Therefore, current recommendation is that SLNB is not required for women 70 years or older with clinically node-negative (T1N0) early-stage hormone receptor-positive and HER2-negative invasive breast who plan to take adjuvant hormonal therapy.

### Invasive Local Recurrence

Ipsilateral breast tumor recurrence (IBTR) after breast conserving surgery or mastectomy occurs in 2–10% of patients after a 10-year follow up [61]. Repeat SLNB in patients with IBTR post-BCT with SLNB has been shown to be feasible. A pooled analysis demonstrated an identification rate of 71.9% with a FNR of 9.4% and accuracy rate of 97.1% [62–64]. As a result, for patients who previously underwent BCT with SLNB and present with clinically node-negative invasive local recurrence, a SLNB is recommended. However, given that all these patients require systemic adjuvant therapy, the utility of SLNB is unclear. Patients who present with clinically node-positive invasive local recurrence require an ALND.

## Conclusion

Breast cancer is a high-volume disease demanding robust research and academic interest, and its management is continually evolving. This evolution has led to evidence-based support for more minimally invasive surgical approaches. While it has taken time, surgeons have largely honored the data endorsing de-escalation techniques when evidence supports noninferiority of survival or quality of life [Table 1]. As a result, treatment has shifted from the disfiguring and low-value radical mastectomy of old to breast conservation surgery. Practice changes that became industry standards based on the foundational works of the NSABP among many others, resulted in a reduction in the extend of in breast resections. Along with this came a reduction in the extend of axillary operations. Axillary surgical practice scaled back from complete axillary clearance to the sentinel lymph node biopsy pioneered in the 1990s. The Z-0011 and AMAROS trials progressed practices even further, as less ALNDs were indicated. In the ongoing quest to remove low-value interventions and reduce chronic complications such as lymphedema, there have been continued evaluation of care in vulnerable groups such as the elderly. Recently implemented limitations to axillary surgery, including complete omission in the elderly population, provide an excellent example of this movement. Consistent efforts to evaluate treatment methods and provide exceptional multi-disciplinary patient care will limit disability, encourage novel practice patterns, and improve survival.

**Table 1:** Selected practice changing studies on axillary management in breast cancer

Year	Landmark Trial	Design	Clinical Significance
2002	Fisher B, Jeong JH, Anderson S, Bryant J, Fisher ER, Wolmark N. Twenty-five-year follow-up of a randomized trial comparing radical mastectomy, total mastectomy, and total mastectomy followed by irradiation. N Engl J Med. 2002 Aug 22;347(8):567–75. https://doi.org/10.1056/NEJMoa020128. PMID: 12,192,016	1665 women with clinically negative axillary treated by radical mastectomy, total mastectomy without axillary dissection but with regional irradiation, or total mastectomy without irradiation plus axillary dissection only if nodes were subsequently positive	NSABP B-04 evaluated the benefit of ALND on survival for patients with palpable, non-fixed, operable tumors in the breast and axilla only No significant differences in disease-free or overall survival were noted between patients who underwent radical mastectomy, total mastectomy, or total mastectomy with regional irradiation
1994	Giuliano AE, et al. Lymphatic mapping and sentinel lymphadenectomy for breast cancer Ann Surg, 220 (1994), pp. 391–398	174 mapping procedures were performed using a vital dye injected at the primary breast cancer site Axillary lymphatics were identified and followed to the first ("sentinel") node, which was selectively excised before ALND	Feasibility of the sentinel lymph node procedure described with intraoperative accuracy of 95.6%
2010	Giuliano AE, McCall L, Beitsch P, et al. Locoregional recurrence after sentinel lymph node dissection with or without axillary dissection in patients with sentinel lymph node metastases: the American College of Surgeons Oncology Group Z0011 randomized trial. Ann Surg 2010;252:426–432	446 clinically node-negative patients who underwent SLNB and had 1 or 2 nodes with metastases were randomized to ALND or no further axillary specific treatment. All patients were treated with lumpectomy and radiation	Patients with cT1-2 breast cancer and 1–2 positive sentinel nodes following breast-conserving surgery with planned whole breast radiation therapy (WBRT) had no overall or disease-free survival benefit when undergoing ALND compared to the observation cohort
2014	Donker M, van Tienhoven G, Straver ME, et al. Radiotherapy or surgery of the axilla after a positive sentinel node in breast cancer (EORTC 10981–22023 AMAROS): a randomised, multicentre, openlabel, phase 3 non-inferiority trial. Lancet Oncol 2014;15:1303–1310	1,425 patients with clinically node-negative cT1-2 primary breast cancer and a positive SN were randomly assigned between ALND and ART	Demonstrated no difference in the 10-year axillary recurrence rate or survival between regional nodal irradiation compared to ALND in a population of cT1/2N + patients with predominantly low volume axillary disease following breast-conserving surgery Included mastectomy patients (17% in each arm) Lower lymphedema risk with radiotherapy observed
2013	Kuehn T, Bauerfeind I, Fehm T, et al. Sentinel-lymph-node biopsy in patients with breast cancer before and after neoadjuvant chemotherapy (SENTINA): a prospective, multicentre cohort study. Lancet Oncol 2013;14:609–618	Four-arm, prospective, multicenter cohort study of women with breast cancer who were scheduled for neoadjuvant chemotherapy. The primary endpoint was accuracy (false-negative rate) of sentinel-lymph-node biopsy after neoadjuvant chemotherapy for patients	The SENTINA trial reported an overall FNR of 14.2% in patients with clinically node positive disease that converted to clinically node negative disease following NAC
2015	Boileau JF, Poirier B, Basik M, et al. Sentinel node biopsy after neoadjuvant chemotherapy in biopsy-proven node-positive breast cancer: the SN FNAC study. J Clin Oncol 2015;33:258–264	Prospective multicentric study of 153 NAC patients with biopsy-proven node-positive breast cancer (T0-3, N1-2) underwent both SLNB and ALND	Post-NAC use of dual tracer, in addition to a targeted axillary dissection which consists of removal of the clipped metastatic node and removal of at least 3 sentinel nodes results in an acceptable false negative rate of 8.4%
2013	Hughes KS, Schnaper LA, Bellon JR, et al.: Lumpectomy plus tamoxifen with or without irradiation in women age 70 years or older with early breast cancer: Long-term follow-up of CALGB 9343. J Clin Oncol 31:2382–2387, 2013	636 women, > 70 years of age, cT1N0M0, estrogen-receptor-positive breast carcinoma treated by lumpectomy, randomized to receive tamoxifen plus radiation therapy (317 women) or tamoxifen alone (319 women)	Low rates of axillary recurrences (3%) were observed Basis for omission of SLNB as recommended by the Society of Surgical Oncology Choosing Wisely guidelines

## Data Availability

No datasets were generated or analysed during the current study.
